# Co-Thermal Oxidation of Lignite and Rice Straw for Synthetization of Composite Humic Substances: Parametric Optimization via Response Surface Methodology

**DOI:** 10.3390/ijerph192416875

**Published:** 2022-12-15

**Authors:** Yanling Li, Xi Chen, Zhen Zhuo, Xueqin Li, Tanglei Sun, Peng Liu, Tingzhou Lei

**Affiliations:** 1 Changzhou Key Laboratory of Biomass Green, Safe & High Value Utilization Technology, Institute of Urban and Rural Mines, Changzhou University, Changzhou 213164, China; 2 Department of Chemical Engineering for Energy Resources, East China University of Science and Technology, Shanghai 200237, China

**Keywords:** lignite, rice straw, humic acid, fulvic acid, response surface methodology

## Abstract

In this study, Baoqing lignite (BL) and rice straw (RS), which were the representatives of low-rank coal and biomass, were co-thermally oxidized to produce composite humic substances (HS), including humic acid (HA) and fulvic acid (FA). Taking HS content as the output response, the co-thermally oxidizing conditions were optimized through single factor experiment and response surface methodology (RSM). The structures of HA and FA prepared under optimized conditions were analyzed by SEM, UV, and FTIR. Results showed that HS content was clearly influenced by the material ratio, oxidation time, and oxidation temperature, as well as their interactions. The optimized co-thermal oxidization condition was as follows: BL and RS pretreated with a material ratio of 0.53, oxidation time of 59.50 min, and oxidation temperature of 75.63 °C. Through verification, the experimental value (62.37%) had a small relative error compared to the predicted value (62.27%), which indicated that the developed models were fit and accurate. The obtained HA had a tightly packed block structure; FA had a loosely spherical shape. The molecular weight of FA was 2487 Da and HA was 20,904 Da; both had a smaller molecular weight than that reported in other literature. FA showed strong bands at 1720 cm^−1^, thus confirming the presence of more oxygen-containing functional groups. The appearance of double peaks at 2900~2980 cm^−1^ indicated that HA contains more aliphatic chains. The co-thermal oxidation of BL and RS gives a new method for the synthesis of HS, and the optimization of co-thermal oxidation conditions will provide fundamental information for the industrialization of composite HS.

## 1. Introduction

Humic substances (HS) are complex mixtures, mainly composed of fulvic acid (FA) and humic acid (HA). FA is soluble in both acidic and alkaline solutions, while HA is soluble in alkaline solutions but insoluble in acidic solutions [1]. HS are formed from animal and plant waste. Through a protracted period of microbial decomposition–transformation and geophysical chemical action, oligomers or polymers are formed with several polar functional groups, such as -COOH, -OH, and C = O [2]. The presence of these groups endows HS with many capabilities, such as acidity, hydrophilia, complexation, adsorptivity, and ion-exchange selectivity [3], which makes HS have great potential in the pharmaceutical [4], agricultural [5,6], industrial [7], and environmental protection [8] applications. Much research has been conducted to transform HS into realistic and technological productivity by the extraction, processing, and modification of HS. For instance, Volikov et al. [9] enhanced surface activity of natural HS with respect to mineral surfaces by functionalization with organosilanes, which was conducted in water. Silanized HS to the soil was contributed to create aggregates, increase soil porosity, and enhance nutrient and water storage capacity. Qin et al. [4] found that in the oxygen-containing functional groups, especially phenolic hydroxyl groups, molecular weight distribution, colloidal properties, and astringency were the material basis of the antidiarrheal activity.

Different composition and storage environments of raw materials cause the variability in HA’s composition, structure, and physical and chemical properties [10]. Therefore, owing to the differences of raw materials and development methods, HS are mainly divided into two types: mineral HS and biochemical HS. Although mineral HS is readily available, the incomplete HS extraction and high HA molecular weight are still problems [5]. In addition to mineral-HS, biochemical-HS also has attracted world-wide attention since the raw material (biomass) of biochemical-HS are renewable and abundant in China. Therefore, this paper proposed that: biomass and lignite were mixed as raw materials and co-thermally oxidated to prepare composite HS, which has high activity and high yield. It is not only an important method for the preparation of biochemical HS but also the highlight of agricultural resources and functionalization.

The chemical pretreatment of raw materials is able to reduce the molecular weight of HS and increase the content of its active functional groups, which can improve the biological activity of HS. Chemical oxidation pretreatment includes hydrogen peroxide activation [11], nitric acid oxidation [12], hydrothermal method [13], and so on. Huang et al. [14] found that nitric acid oxidation was the most effective pretreatment for increasing coal oxidation. This pretreatment method encouraged microbial solubilization in coal, thus increasing the HA yield and the number of oxygen and nitrogen functional groups [15]. Fan et al. [16] used KOH as an activator to extract natural HS and artificial HS from black soil under atmospheric pressure and from tulip leaves/wood chips in a hydrothermal reactor, respectively. The two kinds of HS showed high similarity on chemical structure (abundant aromatic frameworks) and ultimate contents (e.g., N and S elements). L. Aranganathan [17] found that different extractants and extraction conditions would affect the structure and yield of HA. Based on the results, the high yield of HA was obtained from NaOH and KOH treatments and the high elemental “C” level in HA was extracted from NaOH and KOH, whereas high elemental “O” in HA was extracted with Na_4_P_2_O_7_. It can be speculated that the extraction or synthesis conditions have great effects on the physical and chemical properties of the produced HS. Herein, the parametric optimization is vital for the synthesis of HS and its industrialization.

As a common process optimization method, single factor method can only obtain the influence trend of each factor on HS, which cannot reflect the interaction between several variables. Response surface methodology (RSM) is an effective statistical design tool, which generates a set of consecutive experiments to establish the relationships between independent factors and responses. In addition, the function variance and residual and response surface can be calculated directly through software analysis, which has the advantages of cost saving and high credibility. For example, Li [18] used the RSM to optimize the extraction conditions of HA from lignite using Penicillium Ortum MJ51 cell-free filtrate. According to the research [19], RSM was used to determine the optimal conditions, and a mathematical model was established to accurately predict the changes in sludge water content and the extraction rate of HA.

Considering the low yield of biochemical HS, large molecular weight, and low activity of mineral humic acid, this paper presents an effective method for the preparation of composite HS. The mixture of lignite and rice straw was used as raw material, which were pretreated by the co-thermal oxidation method using HNO_3_ solution, then extracted using KOH solution to obtain HS. The pretreatment conditions (including material ratio, liquid–solid ratio, nitric acid concentration, oxidation time, and oxidation temperature were optimized through single factor method and RSM. A mathematical model was established and verified to find the optimal process parameters for preparing HS. The group characteristics of FA and HA products were compared by elemental analysis and functional group analysis. It is expected to provide reference for HS application in industry and agriculture.

## 2. Materials and Methods

### 2.1. Materials

Baoqing lignite (BL) and rice straw (RS) are from Heilongjiang Province and Jiangsu Province, respectively. The raw material was crushed to 60 mesh. The proximate and ultimate analysis of BL and RS are presented in Table 1. The reagents used in the experiment (nitric acid, sodium pyrophosphate, ammonium ferrous sulfate,1, 10-phenanthroline, sulfuric acid, and potassium dichromate) purchased from Sinopharm Chemical Reagent Co., Ltd. were all analytical reagents.

### 2.2. Experimental

#### 2.2.1. Determination of FA and HA Content

**Figure 1:**
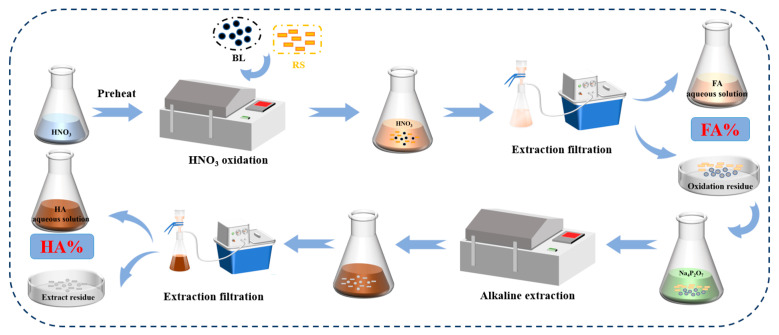
Flow chart of FA and HA content determination.

The detailed testing procedures of FA and HA content are shown in Figure 1. An amount of 0.5 g of the mixture of BL and RS was dissolved in 10 mL of 15% HNO_3_ solution and stirred for 60 min. Then, the mixture was filtered through a Buchner funnel and washed to neutral with distilled water. Additionally, the filtrate was collected to get FA aqueous solution. All of the oxidized residue was transferred to a 250 mL conical flask, and 100 mL of sodium pyrophosphate solution was added and extracted for 2 h at 100 °C. Subsequently, after cooling to room temperature, the filtrate was collected to obtain HA aqueous solution. The contents of FA and HA in aqueous solutions were determined by referring to NY/T 3162-2017 and GB/T1157-2001.

#### 2.2.2. Single Factor Experiment

The FA and HA contents of the co-thermal oxidation of lignite and rice straw were measured during the different pretreatment conditions, and the sum of them was HS content. The ratio of raw materials (BL: RS = 2:1, 1:1, 1:2, 1:2.5, 1:3), HNO_3_ concentration (5~25%), liquid–solid ratio (10~30 mL: g), oxidation temperature (60~100 ℃), and oxidation time (20~90 min) were used as experimental variables to explore the influence of HS of each factor. The three factors with obvious influence on HS were selected as the factors of subsequent response surface experiments, and the center point was determined.

#### 2.2.3. Response Surface Design

Based on results of the single factor experiment, material ratio (RA), oxidation time (TI), and oxidation temperature (TE) had noticeable influence on HS. Develop-Expert V 8.0.6.1 was used to build the Box–Behnken design (BBD) model and design the three-factor and three-level experiments. HS content was taken as the output response, the independent variables were material ratio (1:3, 1:2, 2:3), oxidation time (50, 60, 70 min), and oxidation temperature (70, 80, 90 °C).

#### 2.2.4. Preparation of FA and HA

**Figure 2:**
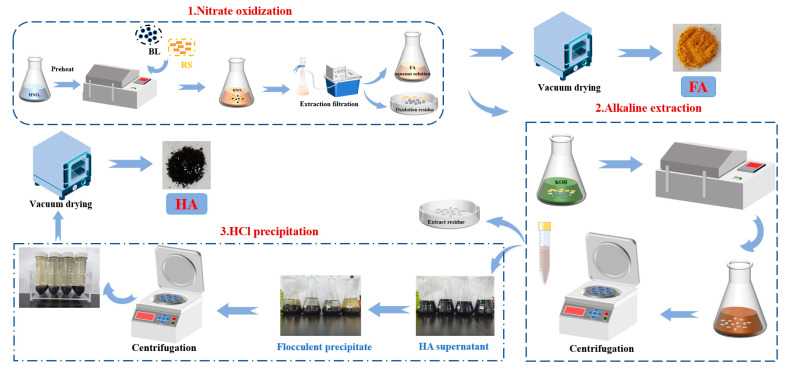
Preparation flow chart of FA and HA products.

The preparation conditions were optimized by RSM. In this part, HA and FA were prepared under the optimized experimental conditions. Different from Section 2.2.1, FA preparation was obtained by vacuum drying the FA aqueous solution. HA was prepared by KOH extraction and HCl precipitation and then dried in a vacuum-drying oven to constant weight. The preparation flow chart of FA and HA products is shown in Figure 2.

### 2.3. Physicochemical Measurements of FA and HA

The morphology and structure of FA and HA products were analyzed by Elements analyzer (Vario EL cube, Elementar, Langenselbold, Germany), ultraviolet-visible spectrophotometer (UV, UV-1900, Shimadzu, Japan), and Fourier transform infrared spectrometer (FTIR, Nicolet IS50, Thermo Fisher, Waltham, MA, USA).

### 2.4. Calculation of FA and HA Contents

The content of FA (*φ_FA_*) in the aqueous solution and the content of HA (*φ_HA_*) in the oxidation residueafter HNO_3_ co-thermal oxidation were determined according to NY/T 3162-2017 and GB/T 11957-2001, respectively. The FA, HA, and HS contents were determined following Equations (1)–(3), respectively.

(1) $$\varphi_{FA}=\frac{3\left(V_{0}-V_{1}\right)c}{R_{c}\times m_{daf}\times 10}\times \frac{a}{b}$$

(2) $$\varphi_{HA}=\frac{3\left(V_{0}-V_{2}\right)c}{R_{c}\times m_{daf}\times 1000}\times \frac{a}{d}\times 100$$

(3) $$\varphi_{HS}=\varphi_{FA}+\varphi_{HA}$$

where *φ_FA_* and *φ_HA_* are the FA and HA contents of raw materials after co-thermal oxidation by HNO_3_, %; *φ_HS_* is total humic acid content, %; *V*_1_ and *V*_2_ are the volumes of standard ammonium ferrous sulfate consumed by the titration test solution, mL; *V*_0_ is the volume of ferrous ammonium sulfate solution consumed by titration blank, mL; *c* is the concentration of ferrous ammonium sulfate standard solution, mol/L; 3 is the equivalent carbon mass to 1 mL standard solution of 1 M (NH_4_)_2_Fe(SO_4_)_2_, g; *R_c_* is the carbon ratio of FA and HA; *m_daf_* is the drying base mass of lignite and rice straw, g; a/b are dilution multiples.

## 3. Results and Discussion

### 3.1. Optimization of FA and HA Preparation Conditions

**Figure 3:**
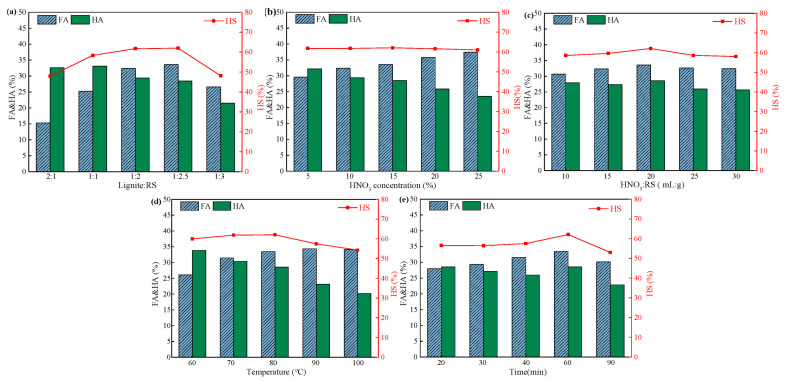
Effects of (**a**) material ratio, (**b**) HNO_3_ concentration, (**c**) ratio of HNO_3_ to rice straw, (**d**) oxidation temperature, and (**e**) oxidation time on FA, HA, and HS contents.

The effects of material ratio, nitric acid concentration, liquid–solid ratio, oxidation time, and oxidation temperature on FA, HA, and HS contents are shown in Figure 3. In Figure 3a, with the increase in RS content in feedstock, FA content showed a trend of first increasing and then decreasing, while the trend of HA content was the opposite. When the material ratio of BL and RS was 1:2 and 1:2.5, HS content was 61.77% and 61.98%, respectively, reflecting a small range of change. However, as the material ratio was adjusted to 1:3, HS content decreased significantly. It was due to this that excessive biomass caused an obvious reduction in the humification of raw materials, which was not conducive to the preparation of HS. In order to save reagents and achieve good economic benefits, 1:2 was chosen as the material ratio for the subsequent study.

According to Figure 3b, there was no significant change in HS content, and FA content was enhanced with the increase in HNO_3_ concentration; this was attributed to the strong oxidation performance of HNO_3_ promoting the decomposition of lignin and hemicellulose in RS and accelerating the oxidation of macromolecular HS into small molecular HS in BL, which consequently results in the decrease in HA content.

In Figure 3c,d, it can be found that the liquid–solid ratio has little influence on FA and HA contents, conversely, the effect of temperature on FA and HA was obvious. It was due to the fact that with the increase in temperature, the oxidation and cracking processes in the raw materials were promoted, the long chain inside the raw materials was broken, and more water-soluble HS was extracted. Accordingly, the amount of HA flocculation precipitation was reduced.

In addition, the effect of oxidation time is shown in Figure 3e. As the oxidation time increased, FA content showed an upward trend. Both FA and HA content reached to the highest when oxidation time was 60 min, and both then displayed a tendency of decline.

The decrease in HS content may be due to the excessive time and high degree of oxidation, which destroyed the structure of BL and RS and converted into low-molecular-weight soluble organic acids and gaseous acid products [20].

According to the results of a single factor experiment, among the five factors, MA, TI, and TE have more obvious influence on HS content. Therefore, these three factors were selected as the variables to further optimize the HNO_3_ pretreatment conditions via RSM. HS content was chosen as the response value, and the optimal values of MA, TI, and TE (1:2, 80 °C, and 60 min, respectively) were taken as the central values of RSM.

### 3.2. RSM Analysis

#### 3.2.1. Experimental Design and Data Processing

On the basis of single factor experimental results, BBD module was used to determine the optimal process conditions for the preparation of HS. BBD is advantageous in that it requires fewer experimental runs to obtain a reasonable optimization. Using the Design-Expert V 8.0.6.1 program, the three factors (MA, TI, and TE) and three levels of experimental design were completed, and the Box–Behnken model was created. The prior single factor test was used to identify the level range of variables. A total of 17 groups of experiments were carried out using HS content as the output response. The design matrix of the variables in coded units is given in Table 2. The test program and results are listed in Table 3.

#### 3.2.2. The establishment of Quadratic Polynomial Regression Model and Regression Analysis

The quadratic model was used to predict the relation between the response (HS content (Y)) and three variables (material ratio (X_RA_), oxidation time (X_TI_), and oxidation temperature (X_TE_). The corresponding empirical equation was presented as Equation (4):

Y = 62.04 + 0.55X_RA_ − 0.13X_TI_ − 0.78X_TE_ + 0.19X_RA_X_TI_ − 0.082X_RA_X_TE_ + 0.26X_TI_X_TE_ − 1.45X_RA_^2^ − 2.05X_TI_^2^ − 0.93X_TE_^2^ (4)

The mathematical function model, Equation (4), was subjected to an analysis of variance (ANOVA) and the statistical significance of the second-order polynomial equation was checked by an F-test. All the corresponding data are reported in Table 4. The F-value of 80.22 was much higher than the standard F-value (F_0.05_ = 4.25) [18], implying a significant level of models and a small chance of noise impacts on their accuracy. *P*-value below 0.05 indicates that the factor had a significant effect on the response values [21], and the *P*-value decreased as the variable’s importance increases. This model’s *P*-value was 0.0001, which meant that it was obviously significant and adequate to describe the response. The order of significance of the three factors was: TE (F = 85.77) > RA (F = 42.75) > TI (F = 2.35).

The mean (Mean) and standard deviation (Std. dev.) were 59.96 and 0.24. The R^2^ value of 0.9904 for HS content demonstrates the accuracy of the model, demonstrating a strong correlation between the predicted and experimental values [22]. This showed that 99.04% of the variation in HS content was covered by the model and only about 0.96% was out of its explaining range [23]. The R^2^_Adj_ (0.9781) was also very high, which implied the higher significance of the model. The R^2^_Pred_ (0.8464) showed a reasonable agreement with the R^2^_Adj_ value of 0.9971. R^2^_adj_ − R^2^_Pred_ = 0.1317, which was less than 0.2, revealing that the model’s reliability and precision were high, and there was a strong correlation between the predicted and actual values. The object of RSM is to detect which experimental parameters generate signals, with a signal-to-noise ratio greater than 4 serving as the reference [24]. The Precision_Adeq_ was 23.8799, which was much higher than 4. Usually, the coefficient of variation (CV%) percentage is a measure of the data’s residual variation relative to the mean. Here, a lower CV% (0.4%) indicated an ideal reliability of the experiment [25]. In addition, the value of PRESS was 6.37, which demonstrated that the model had a high degree of fit. Therefore, the model (Equation (4)) can predict and optimize pretreatment operation conditions.

#### 3.2.3. Reliability Analysis of Model

**Figure 4:**
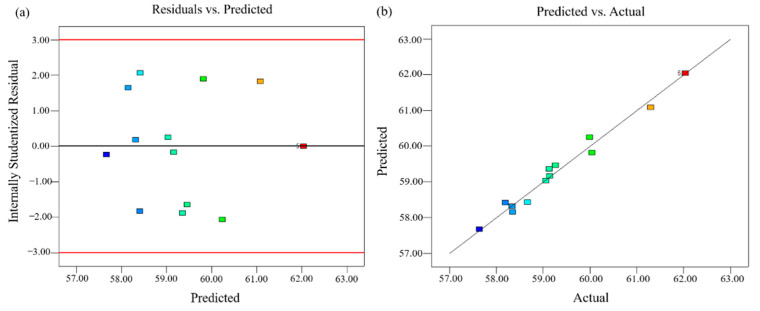
Diagnostics of response surface quadratic model. (**a**) Residual and predicted plot, (**b**) actual and predicted plot.

Through the credibility analysis of the mathematical model (Equation (4)), the residual normal probability distribution of the predicted value and the comparison curve between the predicted value and the actual value can be obtained (Figure 4). As seen from Figure 4a, the residuals were spread evenly on both sides of the line. No data showed a significant departure from the norm, and the errors had a normal distribution, suggesting that the function model was markedly accurate. In Figure 4b, the actual value was near to and spread around the oblique line, which illustrated the experimental values of the model were in good agreement with the expected values.

#### 3.2.4. Interactions among Factors

**Figure 5:**
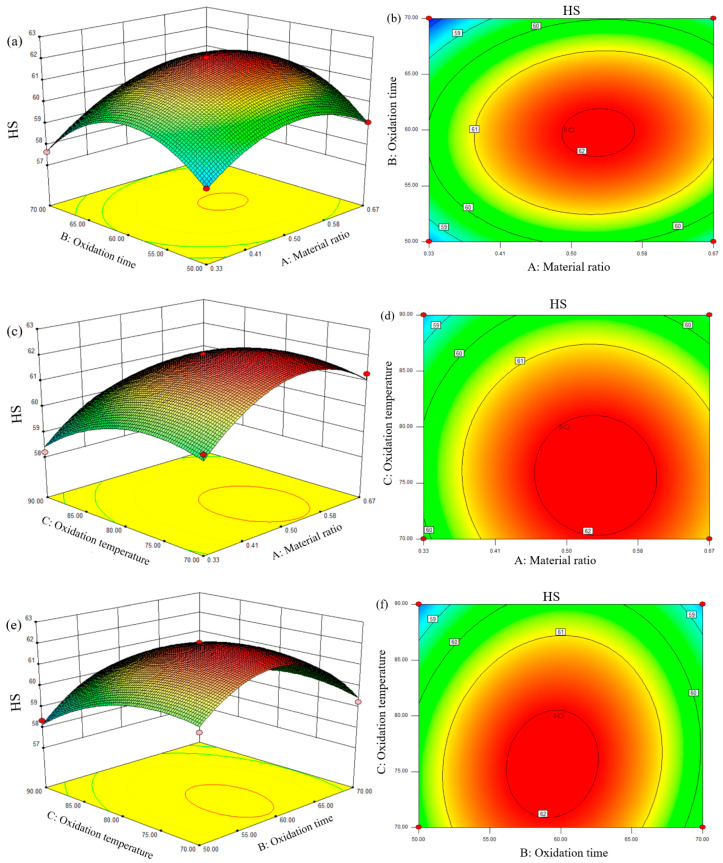
The 3D surface plots and contour plots of different variables’ interaction on HS content (**a**,**b**) oxidation time and material ratio, (**c**,**d**) oxidation temperature and material ratio, (**e**,**f**) oxidation temperature and oxidation time.

Three-dimensional (3D) response surface plots and contour plots were used to further evaluate the interactive impacts of each independent variable on HS content. To determine the ideal pretreatment process parameters for the co-thermal oxidation treatment of BL and RS, the specific influence of each variable on HS content was, therefore, examined based on the response surface diagram of each variable. Results were displayed in Figure 5. HS concentration is shown as a color gradient from red to blue and from high to low. The shape of the contour plot of the response surface can directly reflect the influence of multiple factors on the response value, where the ellipse indicates strong interaction, and the circle is opposite [18]. The response surface function’s shape was expressed by a 3D surface plot. Convex, concave, or saddle-shaped contours suggest a significant interaction between two factors, meaning that the combined effects of the two variables are considerable [26].

As can be seen from the 3D response surface diagram in Figure 5, the images were all raised, demonstrating that there is significant interaction between the two variables. It can be seen from the contour map that the highest HS content was located in the red area. As shown in Figure 5a,b, HS content gradually grew until it reached its peak and then fell as RA and TI increased. The best material ratio was between 0.50 and 0.58, and the best oxidation time was between 57 and 63 min. Figure 5c,d illustrated the interactive effects of TE and RA on HS content. The HS content had a parabolic form as the oxidation temperature to material ratio increases, which was consistent with the *P*-value of AC (0.5160) in the ANOVA (in Table 4). This was supported by the *P*-value of BC (0.0622) in the ANOVA (in Table 4). It was also found from a notably steep slope in Figure 5e,f that there was a significant interaction between TI and TE, and that HS content increased initially with an increase in response time and temperature before dropping.

The results of RSM revealed that the ideal parameters for the highest HS content were as follows: material ratio was 0.53, oxidation time was 59.50 min, oxidation temperature was 75.63 °C, and HS content was 62.27%. In order to verify the effectiveness of the optimization parameters, three parallel experiments were set up. The HS contents of three parallel experiments were 62.20%, 62.17%, and 62.74%, respectively. The average experimental results were 62.37%. The standard deviation was 0.16%, which demonstrated that the model fitted well and could be used to predict the change in HS content during different pretreatment conditions.

### 3.3. Analysis on the Properties of HA and FA

#### 3.3.1. Elemental Analysis of FA and HA

The prepared FA and HA products’ composition was examined by elemental analysis, atomic ratios were calculated to investigate the degree of aromatic condensation and maturity of HS [27], and the results are displayed in Table 5. The elemental compositions of FA and HA clearly differ from one another. FA and HA were mainly composed of carbon and oxygen elements. The carbon and oxygen contents of FA were 28.99% and 65.37%, respectively, while those of HA were 50.98% and 38.88%, which were consistent with the elemental analysis ratio of HA reported in the previous literature [28,29].

The carbon content in HA (50.98%) was much higher than that in FA (28.99%), which was due to the fact that more macromolecular humic acid structures were formed in HA (which were synthesized by KOH extraction and HCl separated out) than in FA (which prepared by direct nitrification). The high hydrogen content in HA (5.65%) can be caused by the new aromatic groups formed and the aliphatic chains broken, or the existence of low lipid group and high aromatic groups. The oxygen content in FA (65.37%) was significantly higher than that in HA (38.88%), which may result from the enrichment of oxygen groups, such as phenols, alcohols, and carboxyl groups, revealing that FA had higher activity than HA. It has been reported that the H/C atomic ratio ranges from 0.7 to 1.5 [30], and the H/C atomic ratios of FA (1.22) and HA (1.33) obtained in this experiment were relatively high in this range, indicating that the condensation or polymerization reaction of FA and HA was obvious. This result may be due to the fact that the co-thermal oxidation process broke the long-chain structure of raw materials and generated volatile small-molecule organic matter. However, HA had less C/N atomic ratio than FA, which may be attributed to the immobilization of nitrogen in the humic process [31]. Equivalently, the thermal decomposition of HNO_3_ in the process of co-thermal oxidation produced -NO_2_, which enters the HA structure in the subsequent alkali extraction process to form stable nitrogen-containing compounds.

#### 3.3.2. SEM Analysis of FA and HA

**Figure 6:**
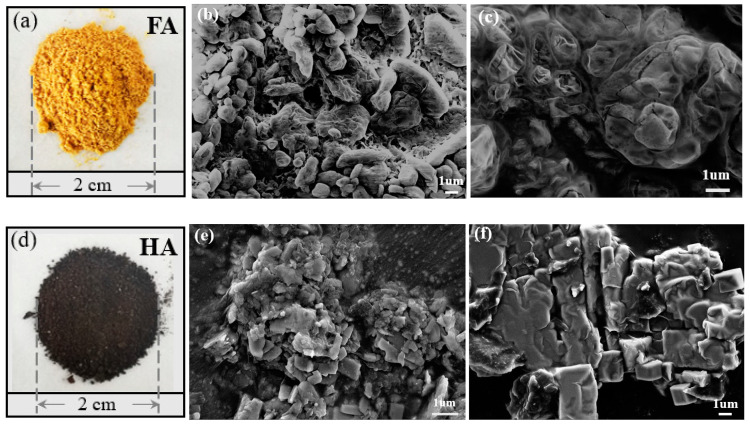
Morphology and SEM images of FA and HA (**a**) FA, (**b**) FA5000, (**c**) FA10000, (**d**) HA, (**e**) HA5000, (**f**) HA10000.

The macro- and micro-morphology of FA and HA products are shown in Figure 6. It can be seen that FA and HA products were obviously different in morphology. FA was orange-yellow, soft, and easy to absorb water in the air. HA was dark brown, with fine particles and greater hardness. According to the SEM images of the products in Figure 6, FA was a mainly fluffy and smooth spherical structure, while HA was dominated by scattered, smooth-edged lumpy structure. In Figure 6b, the spherical particles on the FA surface have obvious gaps and irregular distribution. FA was relatively loose. According to the high magnification diagram of Figure 6c, it can be seen that the surface of spherical particles appeared to have a fold structure, which was more three-dimensional. This loose, globular fold structure may be one of the reasons why FA-absorbed water more easily than HA. Figure 6e,f showed that HA particles were smaller and more irregular than FA particles. The block structure with a smooth surface and flat edges appeared on the surface of HA, which made the product structure more compact. This was consistent with the fact that HA products were harder.

#### 3.3.3. UV-Vis Analysis of FA and HA

UV-Vis spectrophotometer primarily absorbs the quinone, aldehyde, and non-quinone carbonyl groups in FA and HA. Since different raw materials have different conjugate systems, the absorption peaks in UV-Vis region will shift. As the structural parameter of HS, E4/E6 represents its aromatization degree. It has become the foundation for HS analysis in order to predict the relative molecular weight (M) of HA. The M was calculated, as shown in Equation (5):

(5) $$lgM=-0.0893E_{4/6}+5.193$$

The results of UV-Vis test on FA and HA products prepared under the optimized conditions via RSM were shown in Table 6. The maximum absorption peaks of FA appeared near 205 nm, while the maximum absorption peaks of HA appeared at 228 nm, which may be related to the conjugated system with nitrogen of the HNO_3_ solution [32]. E4/E6 in FA (19.750) was significantly larger that in HA (9.773), indicating that FA had smaller molecular weight and stronger activity, but its degree of unsaturated bond conjugation was lower and humification was light. The HA product made by combining lignite and biomass had a smaller M (20,904 Da) than the mineral HA reported in the literature (69,211 Da [33], 148,616 Da [34]), indicating that the addition of biomass increases the conjugated system of lignite and decreases the level of humification.

#### 3.3.4. FTIR Analysis of FA and HA

**Figure 7:**
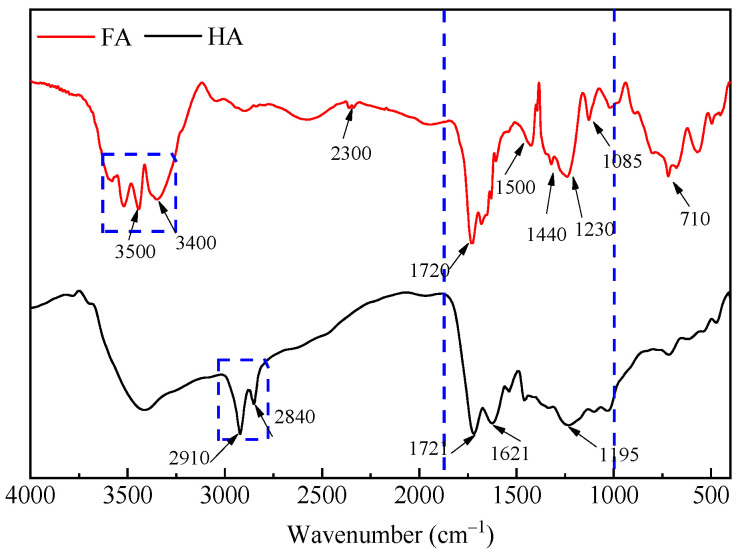
FTIR spectra of FA and HA.

The FTIR spectra of FA and HA extracted from the BL and RS (Figure 7) presented some common and distinctive features. Thiol and sulfo groups had stretching vibration absorption peaks between 500 and 700 cm^−1^ [35]. There were polysaccharides in FA, since the peaks were around 1085~1030 cm^−1^, which were assigned to the C-O stretching vibration of alcohols, ethers, and thiols. At around 1260~1220 cm^−1^, the band was assigned to the -OH and C-O deformation in the carboxyl, phenoxy, and aryl ester [36]. A sharp absorption band at around 1532 cm^−1^ due to -NO_2_ group stretching observed in FA was indicative of -NO_2_ group retention during nitration reaction [37]. At around 1440 cm^−1^, which were the deformation of the aliphatic C-H, the extension of the aromatic C = C bond, and the NH_2_ vibration in the peptide. The FA peak here was stronger than HA, indicating that the branch chain in the raw material was broken by the co-thermal oxidation of HNO_3_. HA showed strong bands at 1621 cm^−1^ (C = C stretching vibrations in olefinic and aromatic compounds) [38]. Both FA and HA showed strong bands at around 1720 cm^−1^, which were the stretching vibration absorption peak of C = O of carboxyl and carbonyl groups (aldehydes and ketones) [39]. The peak strength of FA was significantly higher than that of HA, indicating that FA had more oxygen-containing functional groups and greater activity. This was also consistent with the results of elemental analysis (Table 5). The band at 2330 cm^−1^, peaks for -OH extension, and carboxyl hydrogen bonding could be seen in FA. The double peaks of HA at 2900~2980 cm^−1^ were caused by stretching vibrations of aliphatic -CH, -CH_2_, and -CH_3_ side chain groups of aromatic nuclei, indicating that HA contained more aliphatic chains [36,40]. The stretching vibration of -OH or -NH groups with varying degrees of hydrogen bonding results in the broad and intense FA absorption peak at 3300~3500 cm^−1^ [37,39].

## 4. Conclusions

In this study, RSM was used to optimize the co-thermal oxidation conditions for obtaining FA and HA products. The BBD model took HS content as an output response to obtain the process conditions, and then the morphology, composition, and structure of FA and HA prepared under the optimized conditions were analyzed. The results are as follows:

(1) According to the results of the single factor experiment, the ideal pretreatment conditions for the synthesis of HS were as follows: the material ratio was 0.5, HNO_3_ concentration was 15%, liquid-to-solid ratio was 1:20, oxidation temperature was 80 °C, and oxidation time was 60 min. Under these conditions, HS content was 62.04%. HS contents were clearly influenced by these three parameters of material ratio, oxidation time, and oxidation temperature, which were subsequently chosen as the variables to RSM.

(2) According to the BBD model, TE, TI, and RA all had a significant influence on HS content. The order of significance of the three factors was: TE (F = 85.77) > RA (F = 42.75) > TI (F = 2.35). As can be seen from the 3D response surface diagram, the images were all raised, demonstrating that there was significant interaction between the two variables. The co-thermal oxidation conditions were optimized using RSM as follows: RA was 0.53, TE was 75.63 ℃, TI was 59.50 min, and the experimental HS content under repeated optimum conditions was 62.37%, which was almost equal to the expected value (62.27%) with a low relative inaccuracy of 0.16%, illustrating that the process model was trustworthy.

(3) Under optimized conditions, the obtained HA had a tightly packed block structure, and FA had a loosely spherical shape. The molecular weight of FA was 2487 Da and HA was 20,904 Da, meaning both had smaller molecular weight than that reported in the literature. FT-IR spectra showed that FA had strong bands at around 1720 cm^−1^, thus confirming the presence of more oxygen-containing functional groups than HA. The appearance of double peaks at 2900~2980 cm^−1^ indicated that HA contains more aliphatic chains.

## Figures and Tables

**Table 1:** The proximate and ultimate analysis of raw materials.

Sample	Proximate Analysis *w*/%				Ultimate Analysis *w*_daf_%				
	M_ad_	A_d_	V_daf_	FC_daf_	C_daf_	H_daf_	N_daf_	S_daf_	O_daf_
HL	24.82	21.10	49.75	50.25	72.39	5.50	0.70	0.62	26.29
RS	8.41	12.38	80.91	19.09	42.00	5.41	0.64	0.28	51.67

**Table 2:** The factors and corresponding levels used in the BBD experiment.

Figure 1	Unit	Symbol Used by Model	Coded Factor Level		
			−1	0	1
Material ratio (RA)	g	X_RA_	0.33	0.50	0.67
Oxidation time (TI)	min	X_TI_	50	60	70
Oxidation temperature (TE)	°C	X_TE_	70	80	90

**Table 3:** Experimental scheme and results.

No.	The Mole Ratio of Raw Materials	Time	Temperature	HS Content, %
1	0.67	60	70	61.30
2	0.5	50	90	58.35
3	0.33	50	80	58.34
4	0.5	50	70	60.00
5	0.5	60	80	62.04
6	0.67	60	90	59.13
7	0.5	70	70	59.26
8	0.67	50	80	59.06
9	0.33	60	70	60.04
10	0.5	60	80	62.04
11	0.33	70	80	57.64
12	0.5	60	80	62.04
13	0.5	70	90	58.67
14	0.5	60	80	62.04
15	0.5	60	80	62.04
16	0.33	60	90	58.20
17	0.67	70	80	59.14

**Table 4:** ANOVA of response surface quadratic model for HS content.

Source	Sum of Squares	df	Mean Square	F-Value	*p*-Value-Prob > F	Significant
Model	41.08	9	4.56	80.22	<0.0001	**
A	2.43	1	2.43	42.75	0.0003	*
B	0.13	1	0.13	2.35	0.1692	
C	4.88	1	4.88	85.77	<0.0001	**
AB	0.15	1	0.15	2.59	0.1513	
AC	0.03	1	0.03	0.47	0.5160	
BC	0.28	1	0.28	4.91	0.0622	*
A^2^	8.84	1	8.84	155.45	<0.0001	**
B^2^	17.64	1	17.64	309.98	<0.0001	**
C^2^	3.60	1	3.60	63.34	<0.0001	**
Residual	0.40	7	0.06			
Lack of Fit	0.40	3	0.13			
Pure Error	0.00	4	0.00			
Cor Total	41.47	16				

Std. dev. = 0.24, C. V = 0.4, Mean = 59.96, PRESS = 6.37, R^2^ = 0.9904. Precision_Adeq_ = 23.8799, R^2^_Adj_ = 0.9781, R^2^_Pred_ = 0.8464. Note: A: material ratio, B: oxidation time, C: oxidation temperature, ** very significant, * significant.

**Table 5:** Elemental composition and atomic ratio of FA and HA.

Sample	Elemental Percentage (wt %)					Atomic Ratio		
	C	H	O	N	S	C/N	H/C	O/C
FA	28.99	2.94	65.37	2.24	0.47	15.10	1.22	1.69
HA	50.98	5.65	38.88	4.29	0.20	13.86	1.33	0.57

Note: dry basis.

**Table 6:** UV-Vis data table of FA and HA.

Sample	Λmax(nm)	E_4_	E_6_	E_4_/E_6_	M(Da)
FA	205	0.079	0.004	19.750	2687
HA	228	0.733	0.075	9.773	20,904

## Data Availability

Raw data of this article are available upon request to corresponding author.
